# Nifuroxazide Mitigates Angiogenesis in Ehlrich’s Solid Carcinoma: Molecular Docking, Bioinformatic and Experimental Studies on Inhibition of Il-6/Jak2/Stat3 Signaling

**DOI:** 10.3390/molecules26226858

**Published:** 2021-11-13

**Authors:** Mohamed El-Sherbiny, Rehab M. El-Sayed, Mohamed A. Helal, Afaf T. Ibrahiem, Hoda S. Elmahdi, Mohamed Ahmed Eladl, Shymaa E. Bilay, Asma M. Alshahrani, Mona K. Tawfik, Ziad E. Hamed, Amany O. Mohamed, Sawsan A. Zaitone

**Affiliations:** 1 Department of Basic Medical Sciences, College of Medicine, AlMaarefa University, Riyadh 71666, Saudi Arabia; msharbini@mcst.edu.sa; 2 Anatomy Department, Faculty of Medicine, Mansoura University, Mansoura 35516, Egypt; 3 Department of Pharmacology & Toxicology, Faculty of Pharmacy, Sinai University, El-Arish 45518, Egypt; rehab.mahmoud@su.edu.eg; 4 Biomedical Sciences Program, University of Science and Technology, Zewail City of Science and Technology, October Gardens, 6th of October, Giza 12587, Egypt; Mohamed.hilal@pharm.suez.edu.eg; 5 Medicinal Chemistry Department, Faculty of Pharmacy, Suez Canal University, Ismailia 41522, Egypt; 6 Department of Pathology, Faculty of Medicine, Mansoura University, Mansoura 35516, Egypt; Afaftaha5342@mans.edu.eg (A.T.I.); hoda9_1@mans.edu.eg (H.S.E.); 7 Department of Pathology, Faculty of Medicine, Northern Border University, Arar 9280, Saudi Arabia; 8 Department of Basic Medical Sciences, College of Medicine, University of Sharjah, Sharjah 27272, United Arab Emirates; meladl@sharjah.ac.ae; 9 Department of Biochemistry, Faculty of Pharmacy, Suez Canal University, Ismailia 41522, Egypt; sshawadfy@yahoo.com; 10 Department of Clinical Pharmacy, College of Pharmacy, King Khalid University, Abha 61421, Saudi Arabia; 11 Department of Clinical Pharmacology, Faculty of Medicine, Suez Canal University, Ismailia 41522, Egypt; dmon_kamal@yahoo.com; 12 Elwaha National High School, Arar 73312, Saudi Arabia; ziadehabhamed@gmail.com; 13 Department of Medical Biochemistry, Faculty of Medicine, Assiut University, Assiut 71515, Egypt; amanyosama@yahoo.com; 14 Department of Pharmacology & Toxicology, Faculty of Pharmacy, Suez Canal University, Ismailia 41522, Egypt; 15 Department of Pharmacology & Toxicology, Faculty of Pharmacy, University of Tabuk, Tabuk 71491, Saudi Arabia

**Keywords:** angiogenesis, bioinformatics, Ehrlich’s solid tumors, molecular docking, IL-6/Jak2/STAT3 signaling, nifuroxazide

## Abstract

Nifuroxazide is an antidiarrheal medication that has promising anticancer activity against diverse types of tumors. The present study tested the anticancer activity of nifuroxazide against Ehrlich’s mammary carcinoma grown in vivo. Furthermore, we investigated the effect of nifuroxazide on IL-6/jak2/STAT3 signaling and the possible impact on tumor angiogenesis. The biological study was supported by molecular docking and bioinformatic predictions for the possible effect of nifuroxazide on this signaling pathway. Female albino mice were injected with Ehrlich carcinoma cells to produce Ehrlich’s solid tumors (ESTs). The experimental groups were as follows: EST control, EST + nifuroxazide (5 mg/kg), and EST + nifuroxazide (10 mg/kg). Nifuroxazide was found to reduce tumor masses (730.83 ± 73.19 and 381.42 ± 109.69 mg vs. 1099.5 ± 310.83) and lessen tumor pathologies. Furthermore, nifuroxazide downregulated IL-6, TNF-α, NFk-β, angiostatin, and Jak2 proteins, and it also reduced tumoral VEGF, as indicated by ELISA and immunohistochemical analysis. Furthermore, nifuroxazide dose-dependently downregulated STAT3 phosphorylation (60% and 30% reductions, respectively). Collectively, the current experiment shed light on the antitumor activity of nifuroxazide against mammary solid carcinoma grown in vivo. The antitumor activity was at least partly mediated by inhibition of IL-6/Jak2/STAT3 signaling that affected angiogenesis (low VEGF and high angiostatin) in the EST. Therefore, nifuroxazide might be a promising antitumor medication if appropriate human studies will be conducted.

## 1. Introduction

Cancer and cancer-related morbidity as well as mortality poses a global public health burden. In 2020, 19.3 million new cancer cases were reported, and female breast cancer was the most commonly diagnosed form of cancer worldwide [1]. Signal transducers and activators of transcription (STAT) is known as a transcription factor family that plays key roles in the signaling of cytokine pathogenesis of several cancers [2,3]. Interleukin-6 (IL-6) promotes the phosphorylation of STAT3 protein by janus tyrosine kinase (Jak). STAT3 activation had critical effects on normal cellular processes including cell proliferation and angiogenesis [4,5,6]. This pathway has been previously implicated in tumorigenesis [7,8]. In breast cancer, the Jak/STAT pathway has been shown to be altered [9,10,11]. Jak/STAT3 increases breast cancer stem cells and cancer chemoresistance by the regulation of lipid metabolism. Hence, the inhibition of Jak/STAT3 signaling mitigates the breast cancer stem cells’ identity and the expression of various lipid metabolic genes [12]. In addition, the approach through which IL6/STAT3 regulates breast cancer development is thought to be by promoting Jak and angiogenesis signaling, which has been documented recently [13,14,15].

Following tumor growth, the tumor cells express specific cytokines that regulate intracellular signal transduction and promote the resistance of tumor cells to chemotherapeutic drugs [16,17]. Indeed, angiogenesis is crucial for tumor growth, and the vascular endothelial growth factor (VEGF) has an essential function in tumor development [4]. The abnormal activation of STAT3 signaling is associated with VEGF overexpression [18,19]. Therefore, targeting Jak2/STAT3 signaling is an important therapeutic option in cancer treatment that may help reduce angiogenesis.

Nifuroxazide is an oral nitrofuran antibiotic that is often used as antidiarrheal. Nevertheless, nifuroxazide was shown to be a potent inhibitor of breast cancer growth metastasis through decreasing the count of lung myeloid derived suppressor cells without significant cytotoxicity [20]. Furthermore, it enhances the anti-proliferative activity against colorectal and melanoma cancers [21]. The role of the IL-6/Jak/STAT3 pathway in chemotherapeutic drug-induced cytotoxicity and cell apoptosis has been previously reported [22,23]. Nifuroxazide acts as an effective inhibitor of the STAT3 signaling pathway by reducing Jak2 autophosphorylation in cancer cells [24,25].

Ehrlich’s tumor is an undifferentiated malignant mammary adenocarcinoma in mice [26,27]. The undifferentiated solid form of Ehrlich’s tumor renders this model beneficial in cancer studies, so it had beneficial use for studies of chemotherapy studies and tumor models [28]. These aspects have encouraged attention in finding a novel antitumor agent [29,30].

Although nifuroxazide, an oral antidiarrheal agent, was documented as a STAT3 inhibitor [31], the putative antitumor activity of nifuroxazide against Ehrlich’s solid tumors (ESTs) has not yet been elucidated yet. In addition, the inhibition of IL-6/Jak2/STAT3 signaling was studied as a suggested molecular mechanism that mediates inhibiting angiogenesis. Molecular docking helped to explore the binding mode of nifuroxazide into the SH2 domain of the STAT3 molecule, while bioinformatic study assessed the importance of STAT3 signaling pathways and illustrated key players in its signal transduction toward angiogenesis.

## 2. Results

### 2.1. Molecular Docking Study

**Figure 1:**
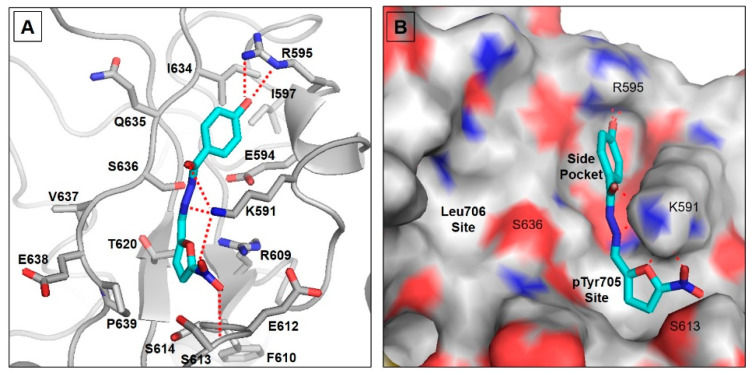
Molecular docking for nifuroxazide. (**A**) Proposed binding model of nifuroxazide in the binding cleft of STAT3. The ligand is displayed as cyan sticks, and the important protein residues are displayed as gray sticks with a cartoon backbone. (**B**) Surface representation of the STAT3 binding cleft showing the three binding pockets discussed.

To analyze the binding mode of nifuroxazide into the SH2 domain of STAT3, we performed molecular docking simulation using the published crystal structure of this transcription factor in complex with DNA (PDB ID: 1BG1) [26]. The STAT3 binding cleft includes three pockets: the pTyr705 binding site, which is polar and basic, the hydrophobic Leu706 site, and a sub-pocket formed by Ile597, Leu607, and Ile634 [32] (Figure 1). Nifuroxazide was predicted to bind into the pTyr705 site, replacing the polar interactions with its nitro group and showing similar polar contacts with Ser613 backbone and Lys591. The latter nicely anchors the ligand by forming H bonds with the carbonyl, the hydrazone nitrogen, and the nitro group of the ligand (Figure 1A). The other side of the compound, the phenyl group, fits into the sub-pocket close to Ile597 and Ile634. It is stabilized in this pocket via a bidentate H bond with Arg595. It is noteworthy that the pTyr705 site is known to be essential for inhibitor binding, and the nitro group of nifuroxazide represents the key binding moiety [33]. However, our data demonstrate that the ligand was able to occupy only two of the three pockets forming the binding cleft (Figure 1B). This could rationalize, at least in part, the suboptimal micromolar potency of this compound toward STAT3. Nevertheless, this compound is a promising lead for repurposing. We can envision an optimized ligand with a third arm that can extend to reach the Leu706 site in a similar fashion to the recently published crystal structure of phosphonic acid derivative SD36 (6NJS) [34].

### 2.2. Bioinformatic Study

**Figure 2:**
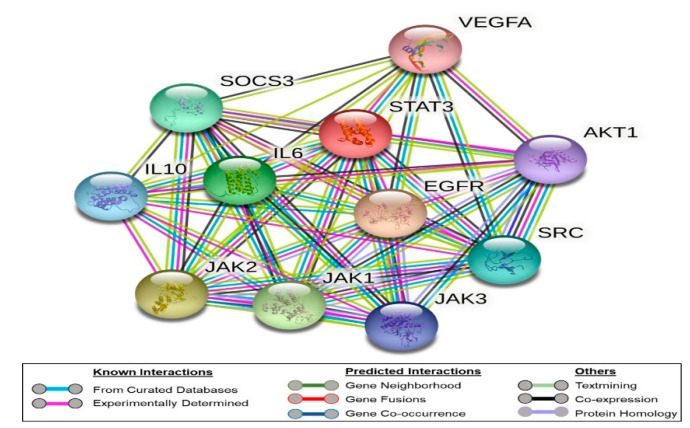
Network of protein–protein interaction as represented by the STRING database. The nodes refer to the proteins under study, while the edges represent the evidence channels used in the prediction. The edges are color-coded, as shown in the legends section of the figure.

To highlight the importance of the STAT3 signaling pathways and illustrate key players in its signal transduction, we performed bioinformatic analysis using the STRING database [34]. It is a comprehensive database of experimentally determined as well as predicted protein–protein interactions, whether they are physical (direct) or indirect ones. In this database, each protein–protein interaction is given a “score” based on seven “evidence channels”. These channels, indicated by colored lines or edges in Figure 2, represent experimental evidence, database citation, gene neighborhood, fusions, or co-occurrence, appearance in literature text, co-expression, and protein homology. Analysis of STAT3 revealed its extensive interaction with VEGFA, EGFR, IL6, JAk1, JAk2, and JAK3 proteins (Figure 2). As shown in Figure 2, the experimental evidence refer to the direct interaction of STAT3 with all of these signaling proteins (pink edges). In addition, the curated databases used in STRING, such as BioGRID, HINT, and APID, refer to the association of these proteins (cyan edges). STRING analysis indicates that STAT3 is co-expressed with JAK1, JAK2, and EGFR.

It is worth noting that as noticed in the STRING analysis, STAT3 is known to relay signals from growth factor receptors in the plasma membrane such as VEGFA to regulate gene expression in the nucleus. We did not observe a direct connection between VEGFA and the JAK3, while the connection with JAK1 and JAK2 is suggested by literature evidence (text mining, green edges) without experimental evidence.

### 2.3. In Vivo Experiment

#### 2.3.1. Ehrlich’s Solid Tumor Model

**Figure 3:**
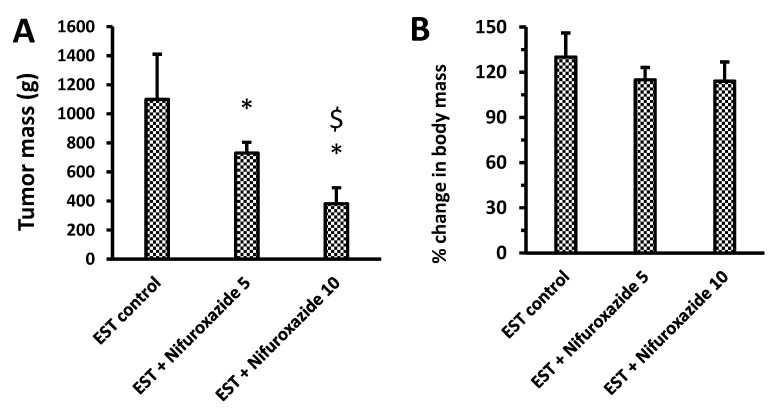
Impact of nifuroxazide treatment on solid tumor masses and percentage change in body masses. (**A**) tumor mass & (**B**) % change in body mass. Data are mean ± SD and evaluated by one-way ANOVA and Bonferroni’s test at *p* < 0.05 (*n* = 6). * Versus the EST control, ^$^ Versus the EST + Nifuroxazide (5 mg/kg) group.

The biggest mass of the grown solid tumors was detected in the EST control group (1099.5 ± 310.83 mg, Figure 3A). Treatment with nifuroxazide (5 or 10 mg/kg) reduced the corresponding tumor mass in a dose-dependent manner, indicating potential antitumor activity (730.83 ± 73.19 and 381.42 ± 109.69 mg, *P* < 0.05, Figure 3A). Calculating the percentage change in body masses in the experimental group revealed non-significant differences among the study groups (Figure 3B).

#### 2.3.2. Relative Gene Expression of Il-6, Jak2, Total STAT3, and VEGF in Solid Tumors

**Figure 4:**
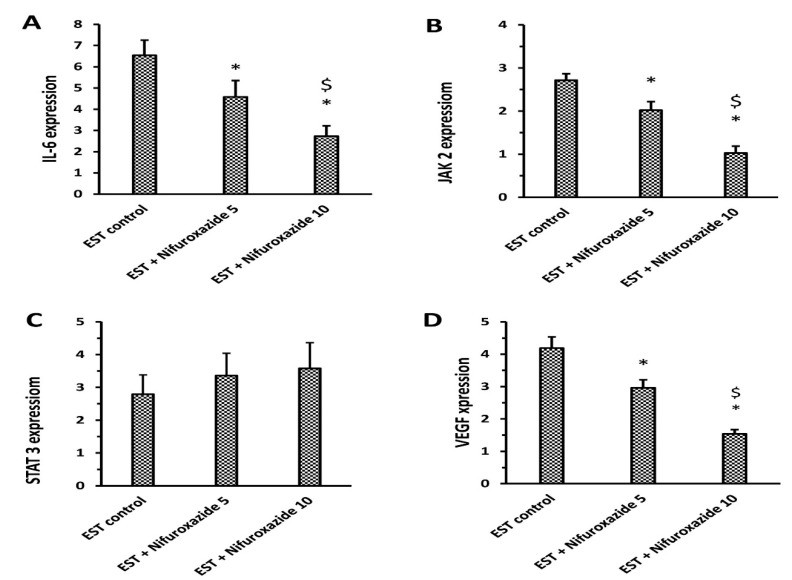
RT-PCR assays for IL-6, JAK2, STAT3, and VEGF in the solid tumors. (**A**) IL-6, (**B**) JAK2, (**C**) STAT3 and (**D**) VEGF expression; Column charts demonstrate data as mean ± S.D., and comparison was achieved by applying one-way ANOVA and Bonferroni’s test at P less than 0.05. * Versus the EST control, ^$^ Versus the EST + Nifuroxazide (5 mg/kg) group.

Treatment with nifuroxazide (5 or 10 mg/kg) significantly downregulated the expression of IL-6, Jak2, and VEGF compared to the EST control group. However, the mRNA expression of total STAT3 was not significantly changed upon treatment with any of the nifuroxazide doses (Figure 4).

#### 2.3.3. Impact of Nifuroxazide on Tumoral Il-6, Jak2, Total STAT3, TNF-α, NF-Kb, VEGF, and Angiostatin

**Figure 5:**
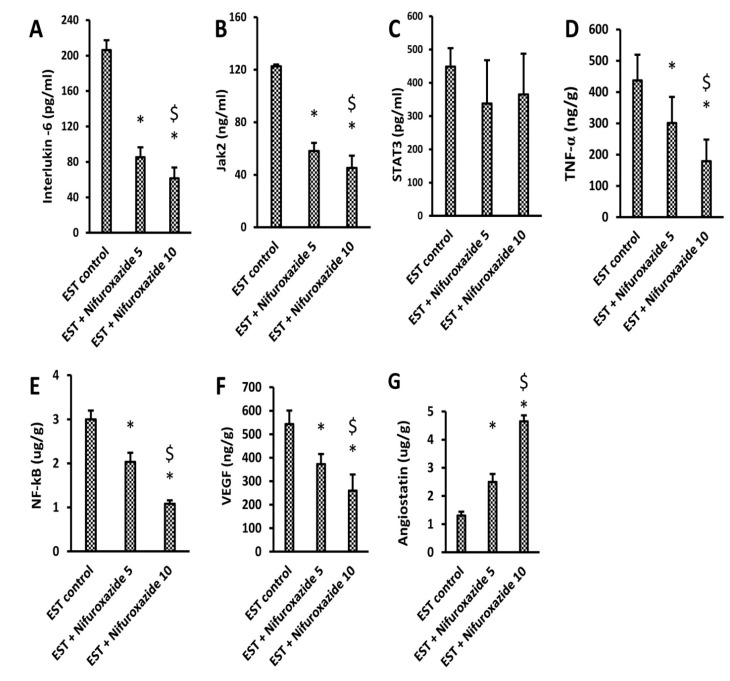
ELISA assays for IL-6, JAK2, STAT3, TNF-α, NF-kB, VEGF, and angiostatin in the solid tumors. (**A**) IL-6, (**B**) JAK2, (**C**) STAT3, (**D**) TNF-α, (**E**) NF-kB, (**F**) VEGF, and (**G**) angiostatin, Column charts demonstrate data as mean ± S.D at P less than 0.05. * Versus the EST control, ^$^ Versus the EST + Nifuroxazide (5 mg/kg) group.

The EST control group showed a high tumoral content of IL-6, Jak2, and STAT3 (Figure 5A–C). The level of IL-6, Jak2, TNF-α, NF-kβ, and VEGF were significantly reduced in the EST + Nifuroxazide (5 and 10 mg/kg) groups in a dose-dependent manner (Figure 5A,B,D–F). Importantly, the intratumoral angiostatin level was increased upon treatment with nifuroxazide (Figure 5G). On the other hand, the total STAT3 level was not significantly reduced by nifuroxazide treatment compared to the EST group (Figure 5C).

#### 2.3.4. Nifuroxazide Downregulated the Phosphorylated STAT3 to Total STAT3

**Figure 6:**
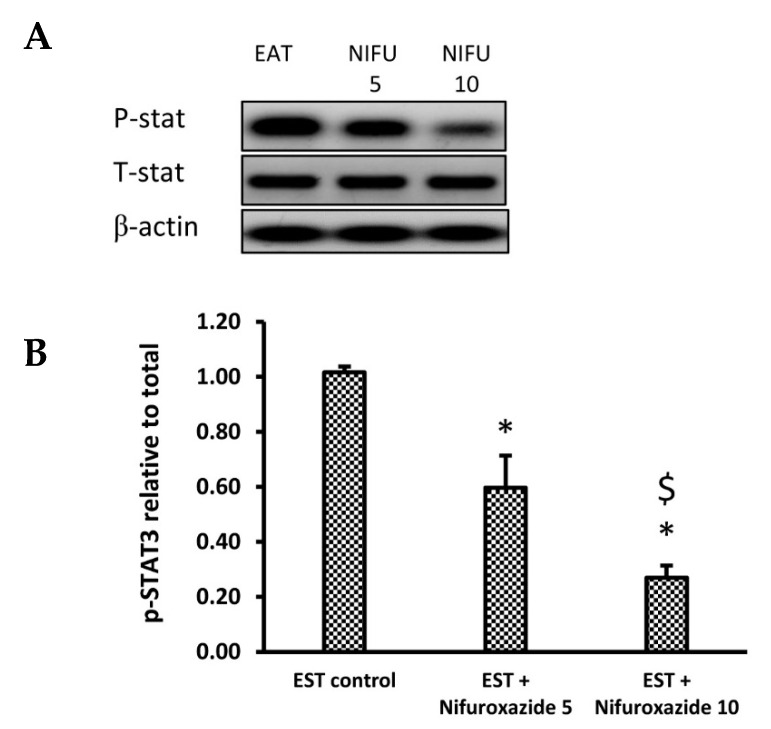
Western blot analysis for phosphorylated and total STAT3. (**A**) photomicrograph representing the Western blot results for the selected proteins in the EST group (**1**), EST + Nifuroxazide (5 mg/kg) (**2**) and EST + Nifuroxazide (5 mg/kg) (3). (**B**) Column chart for phosphorylated STAT3 relative to total STAT3. First, both proteins were normalized to the tumor β–actin level, and then, the relative phosphorylation was calculated. Data are mean ± S.D. and compared at P less than 0.05. * Versus the EST control, ^$^ Versus the EST + Nifuroxazide (5 mg/kg) group.

We used immunoblotting to analyze the impact of nifuroxazide treatment on the phophso-STAT3 (p-STAT3) and total STAT3 (t-STAT3) protein expression. Our results indicated that nifuroxazide treatment (5 or 10 mg/kg) reduced the relative p-STAT3/t-STAT3 in the EST+ nifuroxazide (5 or 10 mg/kg) groups compared to the EST control group (Figure 6A,B). Supplementary Figure S1 shows WB gels for each of the measured markers.

#### 2.3.5. Histopathological Profile of the EST Model

**Figure 7:**
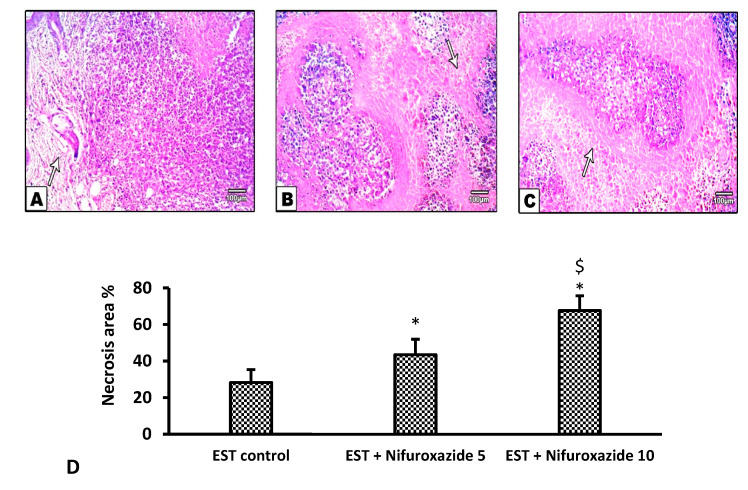
Necrotic areas in the solid Ehrlich tumors stained by hematoxylin and eosin. (**A**) EST control group shows widespread and focal necrotic areas and apoptotic cells, (**B**) EST + Nifuroxazide (5 mg/kg) shows partial necrosis (**C**) EST + Nifuroxazide (10 mg/kg) shows maximal necrosis. (**D**) Column chart representing the percentage of necrotic area, data are mean ± S.D. and compared at P less than 0.05. * Versus the EST control, ^$^ Versus the EST + Nifuroxazide (5 mg/kg) group.

Solid tumors stained with H&E showed different degrees of necrosis among the different experimental groups (Figure 7A–C). Our results demonstrate that nifuroxazide significantly increased the necrotic area in the EST + Nifuroxazide (5 and 10 mg/kg) in a dose-dependent manner Statistical analysis for the measured precise necrosis areas revealed dose-dependent significant increases in the necrosis area by nifuroxazide doses (Figure 7D, *p* < 0.05).

**Figure 8:**
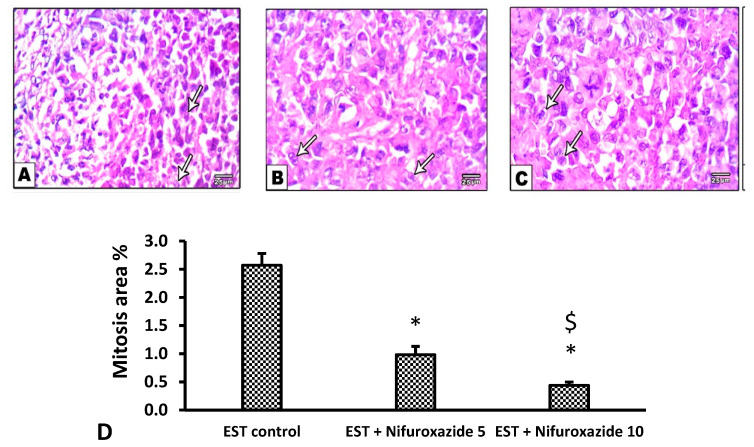
Microscopic pictures of H&E stained sections from tumor masses showing extensive areas of mitosis. (**A**) The EST group shows extensive mitosis (arrows), while a marked reduction in mitosis was observed in the EST + Nifuroxazide 5 or 10 treated groups (**B**,**C**). X: 400, bar 25. (**D**) Column chart representing the percentage of mitotic area, data are mean ± S.D. and compared at P less than 0.05. * Versus the EST control, ^$^ Versus the EST + Nifuroxazide (5 mg/kg) group. EST: Ehrlich solid tumors.

**Figure 9:**
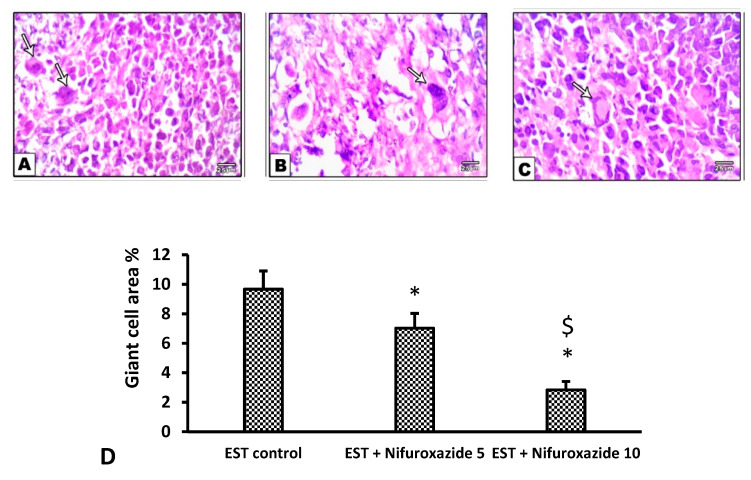
Microscopic pictures of H&E stained sections from tumor masses showing giant cells. Extensive areas of giant cells (arrows) appear in the EST group (**A**), while giant cells were decreased in the EST + Nifuroxazide 5 mg/kg groups (**B**). A marked reduction in the giant area was observed in group treated with EST+Nifuroxazide 10 (**C**). X: 400. (**D**) Column chart for giant cell area percentage, data are mean ± S.D. and compared by one-way ANOVA and Bonferroni’s test at P less than 0.05. * Versus the EST control, ^$^ Versus the EST + Nifuroxazide (5 mg/kg) group. EST: Ehrlich solid tumors.

In the EST control mice, the solid tumors showed pathological manifestations of active tumorigenesis including multiple mitotic spindles and frequent tumor giant cells (Figure 8A and Figure 9A). However, nifuroxazide treatment significantly improved the pathological profile in a dose-dependent manner in the EST + Nifuroxazide (5 or 10 mg/kg) groups showed little appearance for these pathologic manifestations and therefore took lower scores for these parameters (Figure 8B,C and Figure 9B,C, *p* < 0.05).

#### 2.3.6. Immunohistochemical Staining for VEGF in the Solid Tumors

**Figure 10:**
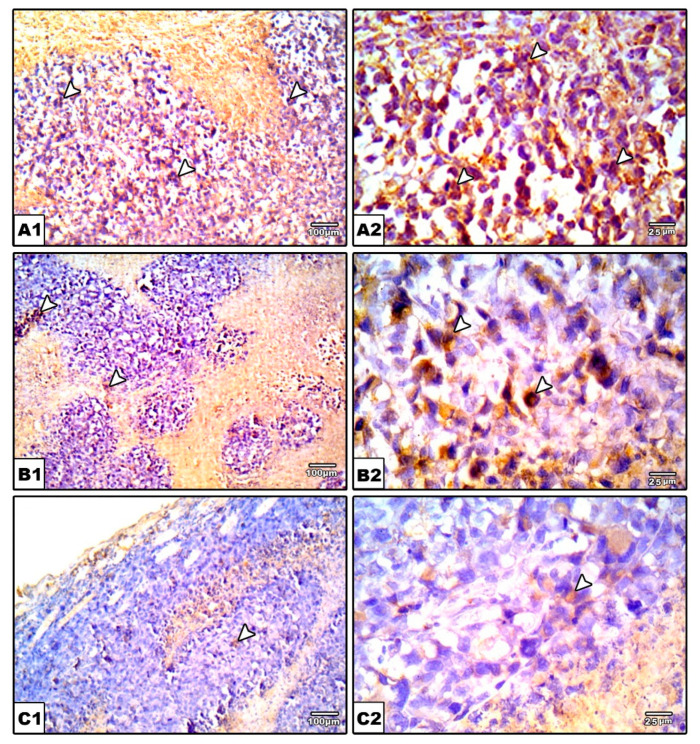

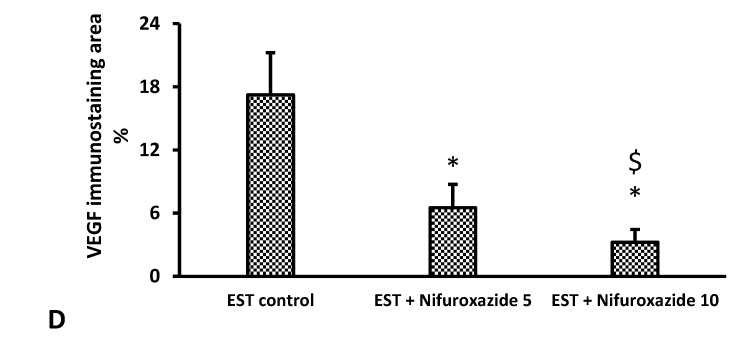
Photomicrographs for sections from tumors immunostained for VGEF. Images show high positive staining in EST (arrow heads) (**A**). A significant reduction in VGEF level was detected in nifuroxazide-treated groups (**B**,**C**) as evidenced by a weaker brown staining (arrow heads). IHC counterstained with Mayer’s hematoxylin. X: 100, bar 100 (**A1**, **B1**, and **C1**), X400 (**A2**, **B2**, and **C2**), bar 25. (**D**) Bar char representing the normalized VEGF immunopositively, data are mean ± S.D. and compared at P less than 0.05. * Versus the EST control, ^$^ Versus the EST + Nifuroxazide (5 mg/kg) group. EST: Ehrlich solid tumors, VEGF: vascular endothelial growth factor.

Our results demonstrate the upregulation of VEGF expression in the EST control group indicating active angiogenesis (Figure 10A). In contrast, nifuroxazide treatment significantly decreased the VEGF immunopositively in the EST + Nifuroxazide (5 or 10 mg/kg) groups showed mild–moderate staining for VEGF (Figure 10B,C). Statistical analysis indicated significant decreases in VEGF immunostaining area in EST + Nifuroxazide (5 or 10 mg/kg) groups compared to the EST control group (*p* ˂ 0.05, Figure 10D).

## 3. Discussion

Despite various therapeutic approaches, 5–11% of breast cancer patients suffer from metastasis [35]; this micro-metastasis is often resistant to systemic therapies. Hence, the development of innovative new medications for preventing tumor growth and metastasis is urgently needed [20]. STAT3 overexpression is closely associated with breast cancer development [36]. In addition, the activation of STAT3 was correlated with poor prognosis in breast cancer patients. Therefore, targeting STAT3 might be beneficial for medicating breast cancer [25]. In the current study, we assumed that the STAT3 inhibitor, nifuroxazide, may suppress the growth of solid Ehrlich carcinomas grown in mice.

In the current study, nifuroxazide was evaluated for its potency against mammary solid tumors in vivo. It was found that nifuroxazide produced a dose-dependent antitumor activity against the growth of EST. Nifuroxazide is a gastrointestinal antibiotic that is mainly utilized for treating infectious colitis and diarrhea [37]. Accumulating evidence suggests the potential anticancer capacity of nifuroxazide through the inhibition of STAT3-dependent gene expression [20,21,38].

The current docking simulation indicated that nifuroxazide was able to occupy only two of the three pockets forming the binding cleft. This could partly explain the suboptimal micromolar potency of this compound toward STAT3. Nevertheless, nifuroxazide can be repurposed. We can predict an optimized ligand with a third arm that can extend to reach the Leu706 site in a similar fashion to the recently published crystal structure of phosphonic acid derivative SD36 (6NJS) [34].

The results obtained from this study demonstrated that nifuroxazide significantly and dose-dependently decreased the tumor mass in the EST model in mice. These results are matched with those obtained previously [25,39]. Similarly, nifuroxazide was reported to produce breast cancer cell apoptosis and prevent pulmonary metastasis in 4T1 cells grown in mice [20] via inhibition of STAT3. Moreover, nifuroxazide was reported to prevent proliferation, cell migration, and encouraged apoptosis in several melanoma cell lines [21]. Again, nifuroxazide has been shown to suppress cell formation, cause apoptosis, and impair the migration and invasion of various colorectal cancer cell types [38], osteosarcoma cells [40], and thyroid papillary carcinoma cells [41]. Furthermore, nifuroxazide was reported to inhibit the STAT3 signaling, which enhances antitumor immunity and reduces colorectal cancer metastasis [38].

The current results demonstrated that nifuroxazide significantly reduced the mRNA expression and protein levels of IL-6, Jak2, and VEGF levels in the solid tumor homogenates. In addition, Western blot analysis indicated that the phosphorylation of STAT3 was reduced significantly in the solid tumors in mice treated with nifuroxazide. The current study confirmed that nifuroxazide lowered the protein level and mRNA expression of IL-6. IL-6 is a cytokine that plays many functions. It is produced by many cells such as epithelial cells, macrophages, dendritic cells, T cells, and B cells [42]. The biological actions of IL-6 are complementary to the immune system, and abnormal IL-6 production is related to the development of various diseases. Furthermore, IL-6 overproduction is thoroughly linked to neoplastic diseases such as sarcoma in Kaposi, myeloma multiple, renal carcinoma, and prostate cancer [43]. As a consequence, inhibiting IL-6 may be helpful in diseases characterized by excessive IL-6 production [44].

Consistently, IL-6 is known to play a crucial role in STAT3 activation in breast cancer [45]. IL-6 activates STAT3 in an autocrine manner through the IL-6 receptor and Jak kinases. Then, the activated STAT3 stimulates the transcription of specific genes that promote the malignant cellular transformation [25]. In different types of human cancers, Jak2/STAT3 signaling is continuously triggered and stimulates tumorigenesis and metastasis by facilitating the expression of cell cycle regulators and angiogenic factors [46,47]. In accordance, targeting STAT3 or its signaling by nifuroxazide has also been documented to cause breast cancer cell apoptosis in 4T1 cells grown in mice [20] via the inhibition of STAT3. Our study is in agreement with a recent study [48] that reported that nifuroxazide reduced colon ulcer in an acetic acid-induced ulcerative colitis model via modulating the IL-6/STAT-3/Wnt axis.

The current study demonstrated that the tumoral VEGF immunopositively was decreased in the nifuroxazide-treated groups. Taken together, it is plausible to speculate that nifuroxazide treatment decreases tumor angiogenesis via the inhibition of the IL-6/Jak2/STAT3 pathway. IL-6, produced by tumors or tumor-infiltrating cells, binds to its target receptor IL-6R and leads to Jak2/STAT3 activation. Then, activated STAT3 is translocated by an importin-dependent mechanism to the nucleus, where it stimulates the target gene transcription. Activated genes include VEGF as well as IL-6. The overexpression of IL-6 creates a positive feedback loop that in turn causes the continuous activity of STAT3 [41]. Since STAT3 phosphorylation inhibition was consistent with inhibiting in vivo breast cancer cell development by nifuroxazide [49], in our opinion, modulation of the Jak2/STAT3 signaling pathway by nifuroxazide declares the availability of drug-combination options that is matched with [50], who studied that phytocompounds target the JAK/STAT signaling pathway for cancer therapy.

Nitazoxanide and tizoxanide are family members to nifuroxazide. Nitazoxanide was reported to suppress the production of IL-6 in stimulated macrophages. In another study, the efficacy of nitazoxanide on IL-1β, IL-6, and TNF-α was assessed in vitro [51]. Tizoxanide was found to decrease cytokine production due to lipopolysaccharide-stimulated macrophages. Furthermore, tizoxanide significantly blocked their genes’ transcription [44].

Previous studies suggested that Jak2 induction of Jak2 activity upstream of STAT3 in breast cancer cells is a crucial step for the estrogen (ER)-dependent progression of cancer [52]. Furthermore, Jak inhibition was proved to be a possible therapeutic option for postmenopausal osteoporosis [53]. Hence, Jak2/STAT3 inhibition by nifuroxazide in mice may be additionally explained by the reduction in the level of ER that contributed to its antineoplastic effect.

Moreover, our results demonstrated that nifuroxazide, a STAT3 inhibitor, increased the intratumoral necrotic areas and downregulated the tumoral VEGF production. In addition, nifuroxazide improved the cancer-induced histopathologic findings, including the spread of giant cells and mitosis picture. Hence, our results provided evidence that ESTs grown in mice produce STAT3, which promotes angiogenesis. Similar to our results, the antitumor effect of nifuroxazide was previously reported in several mouse tumor models including mice bearing A375 melanoma tumors [21], a cancer colon model (CT26 bearing mice) [54], and an orthotopically-implanted hepatocellular carcinoma model [55]; these studies indicated that the antineoplastic effects of nifuroxazide are mediated via inhibiting the STAT3 activity. Moreover, our result, in line with [56], informed that nifuroxazide had potent suppression on STAT3, which provided antitumor and anti-inflammatory effects. Nifuroxazide has also been documented to cause breast cancer apoptosis and prevent pulmonary metastasis in mice via the inhibition of STAT3 [20]. Moreover, nifuroxazide prevented the proliferation, cell migration, and encouraged apoptosis in several melanoma cell lines [21]. Nifuroxazide has been shown to suppress cell formation, cause apoptosis, and impair the migration and invasion of various colorectal cancer cell types [38], osteosarcoma cells [40], and thyroid papillary carcinoma cells [41]

To the best of our knowledge, this is the first report that describes the anti-angiogenic function on nifuroxazide; the current research is the first to study the effect of nifuroxazide on angiogenesis in an EST mammary carcinomas model using VEGF as an angiogenic biomarker. In the same context, nifuroxazide was reported to possess a marked inhibitory effect on the release of VEGF in an orthotopically-implanted hepatocellular carcinoma model. The authors of the previous study concluded that this effect may be related to the lowest expression level of p-STAT3 and the lowest tumor weight [55].

Indeed, a strong association between neovascularization signaling with STAT3 has been previously documented. Activated STAT3 can translocate to the nucleus where it activates the transcription of VEGF mRNA, leading to VEGF upregulation and subsequent angiogenesis [57]. Tumor cells secreting VEGF trigger tumor vessel hyperplasia, promote tumor cell proliferation, and prevent tumor apoptosis [58,59]. In agreement, VEGF was downregulated by chemotherapeutic agents that diminish the tumor growth [60].

Angiogenesis comprises the proliferation, migration, and maturation of vascular endothelial cells. In addition to its importance in the development and wound healing, it is a fundamental mechanism for the tumor growth, being a characteristic feature and hallmark of malignancy [61]. It has a crucial role in pathologic angiogenesis, and its expression is directly associated with poor patient prognosis [62,63]. Therefore, targeting the VEGF is a potential goal during the innovation of novel cancer therapies.

TNF-α was one of the critical mediators of the inflammatory response [64] and angiogenesis response [65,66]. The higher cytokine (TNF-α) level in Ehrlich’s carcinoma-bearing animals might be due to oxidative stress. TNF-α serves as a tumor promoter factor because it encourages cancer cell growth, proliferation, angiogenesis, and metastasis via the NFkB signaling pathway [67,68].

Angiostatin is a naturally occurring endogenous angiogenesis inhibitor found in humans and numerous other animal species. It is a plasminogen proteolytic fragment recovered from tumor-bearing mice [69]. Angiostatin decreases tumor metastasis by reducing blood vessel creation and is thought to restrict endothelial cell migration and prevent tumor growth [70]. As a result, a thorough knowledge of the angiogenic pathway offers the possibility of yielding effective therapies for the advancement of cancer therapies.

More than 300 angiogenic inhibitors have been identified up to now, with more than 80 medicines generated from them in various stages of clinical development [71,72]. Vanucizumab and nesvacumab successfully suppressed tumor growth and demonstrated anti-angiogenic effects [73,74,75,76]. Moreover, in the Ehrlich ascites carcinoma model, an acridine derivative exerts anti-angiogenic effects and cell cycle arrest [77]. So, to suppress tumor formation, innovative angiogenic therapeutic targets must be identified, as well as novel medications developed as alternative or in combination with existing therapies. According to previous evidence [21,38,78], angiogenesis can be inhibited by blocking STAT3 inhibitors by nifuroxazide.

The safety of nifuroxazide was reported previously in mice bearing tumors when the authors did not observe any prominent adverse effects such as toxic death or loss in body weight. The authors declared that there was no blood system abnormalities nor identified pathology in various organs including the liver, the heart, the spleen, and the kidney by microscopic examination in mice that received nifuroxazide therapy; they also excluded any possible elevations in liver enzymes or serum creatinine levels or blood cells [20]. Consistently, the safety of nifuroxazide was reported in human [24]. Certainly, nifuroxazide is a relatively old medication with well-known safety; hence, the current study offers a novel opportunity and therapeutic venue for counteracting solid mammary carcinomas utilizing nifuroxazide.

## 4. Materials and Methods

### 4.1. Chemicals

Nifuroxazide (Batch #1603105414) was obtained in form of a canary yellow powder from Hikma Pharma (6th of October City, Egypt); it was administrated as oral suspension in 0.5 % carboxymethylcellulose.

### 4.2. Molecular Docking

Nifuroxazide was sketched in the Molecular Operating Environment (MOE; ver. 2014) and minimized using the MMFF94 force field with the default cutoff and a distance-dependent dielectric constant to a gradient of 0.001 kcal/mol/Å2 [55]. The 2.25 Å crystal structure of STAT3 in complex with DNA (PDB ID: 1BG1) was used for the docking simulation. The protein was first processed using the “Protein Preparation” wizard and the “Protonate 3D” tool within the MOE software to add hydrogens and correct any errors in the structure. Then, the minimized ligand was docked into the putative binding cleft of STAT3 utilizing the Dock module in MOE software. Specification of the binding site was performed using the side chain of Arg609 within the critical pTyr705 binding site. Docking was achieved by the default settings and the Triangular Matcher method. Docking poses were visually examined by Pymol graphical software (Version 2.0, Schrodinger, LLC, NY, USA) [79].

### 4.3. Bioinformatics Study

The STRING database (https://string-db.org, accessed on 1 October 2021) was employed to simulate the interaction network of STAT3 with other cellular proteins. Briefly, we selected “Protein by Name”, Homo sapiens as the organism, and performed the search. The “network” of nodes represents the proteins connected by edges, thus referring to the seven channels of evidence linking these proteins. These edges are color-coded to denote each element of evidence used to determine the connection. We used the default settings of a median confidence level (0.400) and a Max Number of Interactors to Show of 10 in the first shell only.

### 4.4. In Vivo Antitumor Activity against Solid Ehrlich’s Tumors

#### 4.4.1. Animals

Twenty-one female Swiss albino mice, with body weight range 18–26 g, were housed in polyethylene cages at temperature equals 25 ± 5 °C and normal light/dark cycles with food and water available ad libitum. The mice were allowed 11 days to acclimatize. The animal experiment was approved by the Institutional Research Ethics Committee (Approval # 201906RA4).

#### 4.4.2. Ehrlich’s Ascites Carcinoma Cell Line and Tumor Inoculation in Female Mice

Ehrlich’s carcinoma cells were obtained from by the National Cancer Institute in Cairo. Before inoculation, counting of the Ehrlich’s ascites carcinoma cells was done using a Trypan blue exclusion test [80]. Following acclimatization, the abdominal fur was shaved, and 0.2 mL of Ehrlich cells (2.5 × 106 cells) were inoculated bilaterally at the top ventral mammary sites to form ESTs [81].

#### 4.4.3. Experimental Design

The mice were allocated into groups (7 mice per group). Group 1 included the EST control group in which mice served as the untreated positive control group, as designed in previous studies [29,82,83]. Groups 2 and 3 included the nifuroxazide-treated mice (5 and 10 mg/kg), in which mice with ESTs received nifuroxazide treatment (5 and 10 mg per kg, respectively) by an oral gavage tube for 3 weeks starting from day 8 after tumor inoculation. The doses of nifuroxazide were selected based on previous studies [31,84]. The dose range for the antitumor effect of nifuroxazide in rodents ranges from 25 to 50 mg/kg for 12 days for oral use [38] or about 10 mg/kg for injection for 7 days [55] or 10 and 50 mg/kg for injection for 7 days in mice [20].

The usual human dose for nifuroxazide is 200 mg x 4 times a day for 3 days. In the present study, nifuroxazide (5 and 10 mg/kg) was used, and this can be translated to the human equivalent dose by using the Reagan–Shaw method [85]. According to the formula, the human equivalent dose (mg/kg) = animal dose (mg/kg) 6 animal (km)/ human (km). Km for human adults is 37, while for a 20 g mouse, it is 3. Thus, the human equivalent of murine dose of 5 and 10 mg/kg are 28.35 and 56.7 mg for an average size of a 70 kg adult human. Therefore, all the selected doses in the present study are still below the maximum dose for humans.

#### 4.4.4. Sample Collection

After finishing the experiment, mice were weighed and the percentage of increase in body mass was calculated. Then, mice were sacrificed by cervical dislocation under anesthesia. The two tumor discs were dissected from each mouse, and their weight was recorded. The right tumor disks were fixed in phosphate-buffered formalin for 18–24 h at room temperature for histopathology and immunohistochemistry. The left tumor disks were immediately frozen at −80 °C for subsequent assays.

#### 4.4.5. Relative Gene Expression

In order to investigate the relative expression of IL-6, Jak2, STAT3, and VEGF, RNA was extracted using Promega SV Total RNA Isolation System (Madison, WI, USA) and reverse transcribed with high-capacity cDNA Reverse Transcription Kit (Applied Biosystems, Waltham, MA, USA) according to the manufacturer’s protocol. Real-time PCR was performed using a SYBR Green PCR Master mix (Fermentas, Waltham, MA, Applied Biosystems, USA). The relative expression of each target gene was determined by the comparative threshold method (2-ΔΔCt) normalized to the housekeeping gene glyceraldehyde 3-phosphate dehydrogenase (GAPDH) [54,86]. Primer sequences are detailed in Table 1.

#### 4.4.6. Assessment of IL-6, Jak2, STAT3, and VEGF

Tumor pieces were homogenized in RIPA buffer using Teflon homogenizer on ice (ART-MICCRA D-S, Heitersheim, 79423, Germany), and homogenates were cleared via centrifuging at 3000 rpm for 10 min (Sigma 3K30, Sigma-Aldrich Chemie Gmbh). IL-6, Jak2, STAT3, TNF-α, NF-kB, VEGF, and angiostatin were assessed in the tumor lysates by ELISA using the following kits (IL-6 (cat# EK0411, Boster Biological Technology, Pleasanton, CA, USA), Jak2 (cat# MBS7251994, MyBiosource, Shanghai, China), total STAT3 (cat# MBS2500929, MyBiosource, Shanghai, China), TNF-α (SEA133Mu, Cloud-Clone Corp., Fernhurst Dr., Katy, TX, USA), NF-kB (SEB824Mu, Cloud-Clone Corp., Fernhurst Dr., Katy, TX, USA), VEGF (cat# SEA143Mu, Cloud Cone Corp., Katy, TX, USA) and angiostatin (LS-F6507, LS Bio, Seattle, WA, USA) on an automated ELISA reader at 450 nm (Stat Fax 2100, Ramsey, MN, USA).

#### 4.4.7. Western Blot Analysis for p-STAT3 and Total STAT3

Total proteins were extracted from frozen tumors using RIPA buffer supplemented with protease inhibitor. Then, proteins were resolved on a SDS-PAGE and moved to polyvinylidene difluoride (PVDF) membrane [87]. Blocking of the membranes was achieved by using 3% bovine serum albumin in Tris-buffered saline with Tween 20 (TBST80) 60 min at room temperature. Next, membranes were incubated in primary mouse specific rabbit monoclonal antibodies against STAT3 (#4904, 1:2000), rabbit monoclonal antibodies against P-STAT3 (#9145, dilution 1:2000), and mouse monoclonal antibodies against β-actin (#3700, dilution 1:1000) from Cell signaling technology, Danvers, MA, USA) overnight at 4 °C. Then, membranes were incubated with horseradish peroxidase-conjugated secondary antibody (Novus Biologicals, Centennial, CO, USA). Blots were detected with a chemiluminescent kit (Bio-Rad catalog #170-5060) and imaged (ImageQuantTMLAS500, GE Healthcare Life Sciences). The intensity of target proteins was normalized to the β-actin protein using Image-J1.52p (NIH, Bethesda, MD, USA).

#### 4.4.8. Tumor Pathology

The formalin fixed paraffin-embedded (FFPE) tumor specimens were sectioned at 4 μm, stained by hematoxylin and eosin (H + E), and examined by a light microscope [82,88]. Analysis was carried out by ImageJ (NIH, Bethesda, MD 20814, USA).

#### 4.4.9. Immunohistochemistry for Vascular Endothelial Growth Factor

Tumor sections (4 μm thickness) were deparaffinized in xylene and rehydrated in descending ethanol series. Following antigen retrieval, sections of rabbit polyclonal anti-VEGF antibody (diluted as 1:500, cat# GTX102643, Gene Tex, Irvine, CA, USA) were kept for 16 h at 4 °C and a secondary biotinylated antibody was kept for 1 h. The immune interaction was visualized by Power-Stain™ 1.0 Double Stain kit I (Genemed Biotechnologies, San Francisco, CA, USA) following the manufacturer’s protocol and counterstained with Mayer’s hematoxylin. Negative control sections were prepared by adding the primary antibody with phosphate-buffered saline. Photomicrographs were taken at x 10 and x 40 magnification by an optical Olympus microscope (Tokyo, Japan) coupled to PC-driven digital camera (Olympus E-620). Image quantification of the 10x images was achieved by the ImageJ MacBiophotonics program (NIH, Bethesda, MD, USA).

### 4.5. Statistical Analysis

Data were collected and presented as mean ± standard deviation (S.D.). The difference between variables was assessed by one-way analysis of variance (ANOVA), and the Bonferroni’s test for pair-wise group comparison. Results were analyzed by version 19 of SPSS (SPSS Inc., ver 19, Chicago, IL, USA) and tested for the possibility of Gaussian distribution by applying the K-S test. The significance level was fixed at *p* < 0.05.

## 5. Conclusions

In brief, we have shown that nifuroxazide therapy significantly reduced mammary solid tumors grown in mice via inhibiting angiogenesis; this was—at least partly—mediated by the inhibition of IL-6/Jak2/STAT3 signaling. In fact, nifuroxazide was able to occupy only two of the three pockets forming the binding cleft. Therefore, it would be beneficial to alter the nifuroxazide structure to occupy all three pockets in STAT3 to develop a more potent derivative. Taken together, nifuroxazide might be a promising treatment for mammary carcinomas.

## Figures and Tables

**Table 1:** Primer sequence for the selected genes.

Primer		Sequence
**GAPDH**	Forward	5′-CATCACTGCCACCCAGAAGACTG-3′
	Reverse	5′- ATGCCAGTGAGCTTCCCGTTCAG-3′
**IL-6**	Forward	5′-TACCACTTCACAAGTCGGAGGC-3′
	Reverse	5′-CTGCAAGTGCATCATCGTTGTTC-3′
**Jak2**	Forward	5′-GCTACCAGATGGAAACTGTGCG-3′
	Reverse	5′-GCCTCTGTAATGTTGGTGAGATC-3′
**STAT3**	Forward	5′-AGGAGTCTAACAACGGCAGCCT-3′
	Reverse	5′-GTGGTACACCTCAGTCTCGAAG-3′
**VEGF**	Forward	5′-CAGGCTGCTCTAACGATGAA-3′
	Reverse	5′-CAGGAATCCCAGAAACAACC-3′

## Data Availability

Data for this study are availability from the authors upon request.

## Supplementary Materials

The following are available online, Figure S1. Western blot gels for the measured proteins.

## Sample Availability

Samples of the compounds are not available from the authors.
