# Supplementary material for: Nifuroxazide Mitigates Angiogenesis in Ehlrich’s Solid Carcinoma: Molecular Docking, Bioinformatic and Experimental Studies on Inhibition of Il-6/Jak2/Stat3 Signaling
Source: Molecules. 2021 Nov 13;26(22):6858. doi: 10.3390/molecules26226858 (PMC8621155; doi:10.3390/molecules26226858)
Supplement: Supplementary file 1 [file molecules-26-06858-s001.zip › molecules-1427390-supplementary.pdf]

molecules

Supplementary File

Nifuroxazide Mitigates Angiogenesis in Ehlrich's Solid Carcinoma: Molecular Docking, Bioinformatic and Experimental Studies on Inhibition of Il-6/Jak2/Stat3 Signaling

Mohamed El-Sherbiny [1,2], Rehab M. El-Sayed [3,†], Mohamed A. Helal [4,5], Afaf T. Ibrahiem [6,7], Hoda S. Elmahdi [6], Mohamed Ahmed Eladl [8], Shymaa E. Bilay [9], Asma M. Alshahrani [10,*], Mona K. Tawfik [11], Ziad E. Hamed [12], Amany O. Mohamed [13] and Sawsan A. Zaitone [14,15,*,†]

1 Department of Basic Medical Sciences, College of Medicine, AlMaarefa University, Riyadh 71666, Saudi Arabia; msharbini@mcst.edu.sa
2 Anatomy Department, Faculty of Medicine, Mansoura University, Mansoura 35516, Egypt
3 Department of Pharmacology & Toxicology, Faculty of Pharmacy, Sinai University, El-Arish 45518, Egypt; rehab.mahmoud@su.edu.eg
4 Biomedical Sciences Program, University of Science and Technology, Zewail City of Science and Technology, October Gardens, 6th of October, Giza 12587, Egypt; Mohamed.hilal@pharm.suez.edu.eg
5 Medicinal Chemistry Department, Faculty of Pharmacy, Suez Canal University, Ismailia 41522, Egypt
6 Department of Pathology, Faculty of Medicine, Mansoura University, Mansoura 35516, Egypt; Afaftaha5342@mans.edu.eg (A.T.I.); hoda9_1@mans.edu.eg (H.S.E.)
7 Department of Pathology, Faculty of Medicine, Northern Border University, Arar 9280, KSA
8 Department of Basic Medical Sciences, College of Medicine, University of Sharjah, Sharjah 27272, UAE; meladl@sharjah.ac.ae
9 Department of Biochemistry, Faculty of Pharmacy, Suez Canal University, Ismailia 41522, Egypt; sshawadfy@yahoo.com
10 Department of Clinical Pharmacy, College of Pharmacy, King Khalid University, Abha 61421, KSA; Ashahrany@kku.edu.sa
11 Department of Clinical Pharmacology, Faculty of Medicine, Suez Canal University, Ismailia 41522, Egypt; dmon_kamal@yahoo.com
12 Elwaha national high school, Arar 73312, Northern borders region, Saudi Arabia; ziadehabhamed@gmail.com
13 Department of Medical Biochemistry, Faculty of Medicine, Assiut University, Assiut 71515, Egypt; amanyosama@yahoo.com
14 Department of Pharmacology & Toxicology, Faculty of Pharmacy, Suez Canal University, Ismailia 41522, Egypt; Sawsan_zaytoon@pharm.suez.edu.eg
15 Department of Pharmacology & Toxicology, Faculty of Pharmacy, University of Tabuk, Tabuk 71491, Saudi Arabia
† The authors contributed equally to this manuscript
* Correspondence: Sawsan_zaytoon@pharm.suez.edu.eg (S.A.Z.); ashahrany@kku.edu.sa (A.M.A.)

P-STAT 3	t-STAT3	B-actin
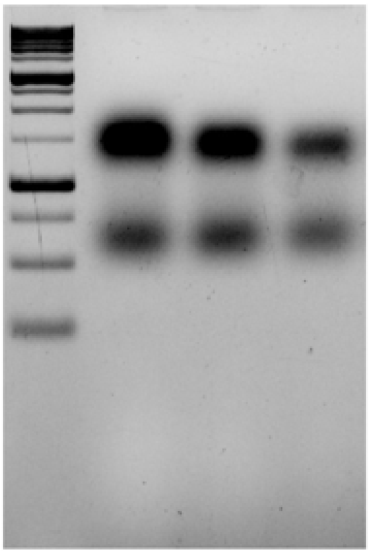	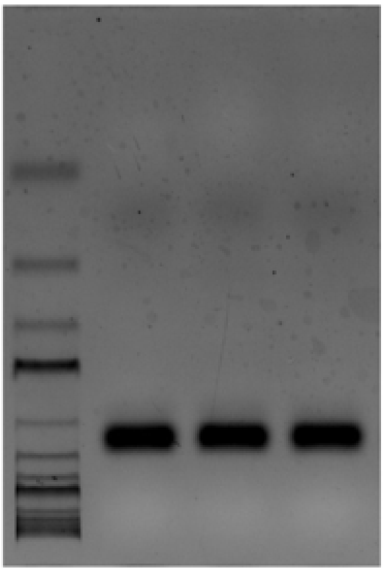	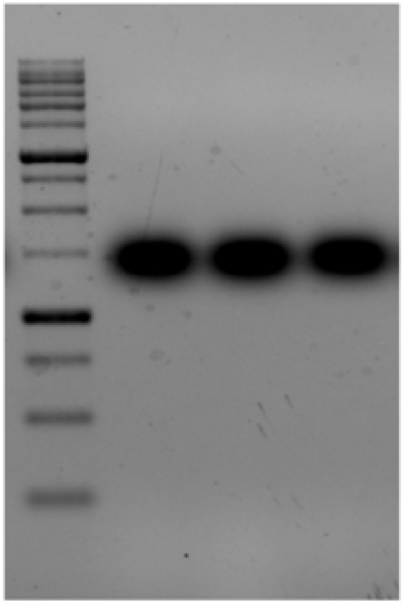

Figure S1. Western blot gels for the measured proteins.
